# Development of a Low-Cost Wearable Data Glove for Capturing Finger Joint Angles

**DOI:** 10.3390/mi12070771

**Published:** 2021-06-30

**Authors:** Changcheng Wu, Keer Wang, Qingqing Cao, Fei Fei, Dehua Yang, Xiong Lu, Baoguo Xu, Hong Zeng, Aiguo Song

**Affiliations:** 1 College of Automation Engineering, Nanjing University of Aeronautics and Astronautics, Nanjing 211100, China; keerwang2021@163.com (K.W.); fei.fei@nuaa.edu.cn (F.F.); dhyang@nuaa.edu.cn (D.Y.); luxiong@nuaa.edu.cn (X.L.); 2 School of Instrument Science and Engineering, Southeast University, Nanjing 210096, China; xubaoguo@seu.edu.cn (B.X.); hzeng@seu.edu.cn (H.Z.); a.g.song@seu.edu.cn (A.S.); 3 School of Aviation Engineering, Nanjing Vocational University of Industry Technology, Nanjing 210023, China; caoqingqing87@gmail.com

**Keywords:** data glove, grating strip, finger joint angle

## Abstract

Capturing finger joint angle information has important applications in human–computer interaction and hand function evaluation. In this paper, a novel wearable data glove is proposed for capturing finger joint angles. A sensing unit based on a grating strip and an optical detector is specially designed for finger joint angle measurement. To measure the angles of finger joints, 14 sensing units are arranged on the back of the glove. There is a sensing unit on the back of each of the middle phalange, proximal phalange, and metacarpal of each finger, except for the thumb. For the thumb, two sensing units are distributed on the back of the proximal phalange and metacarpal, respectively. Sensing unit response tests and calibration experiments are conducted to evaluate the feasibility of using the designed sensing unit for finger joint measurement. Experimental results of calibration show that the comprehensive precision of measuring the joint angle of a wooden finger model is 1.67%. Grasping tests and static digital gesture recognition experiments are conducted to evaluate the performance of the designed glove. We achieve a recognition accuracy of 99% by using the designed glove and a generalized regression neural network (GRNN). These preliminary experimental results indicate that the designed data glove is effective in capturing finger joint angles.

## 1. Introduction

Human hands are closely related to our life. It is one of the most frequently used body parts for human interaction with the outside world. Basic hand motions include grasping, pinching, and stretching. The combination of these motions helps to complete a large number of tasks in everyday life. At present, monitoring and tracking of hand motions and application of these motions to human-computer interaction, rehabilitation training, sign language recognition, and teleoperation have attracted considerable attention from researchers.

In recent decades, many hand motion monitoring and tracking systems based on vision technology, exoskeleton mechanisms, and data gloves have been investigated. In particular, wearable data gloves have attracted extensive attention because they are convenient to wear, free from environmental influences and do not restrict the user’s hand motions. Researchers have developed a large number of commercial data gloves and prototypes based on bending resistors, inertial measurement units (IMUs), optical fiber sensors, and more [1,2,3,4].

The bending resistor is a flexible strip-shaped sensor whose resistance changes with the bending of the sensor. Finger joint angles can be measured by placing several bending resistors on the back of a fabric glove. Wu et al. developed a bending resistance based data glove and used it for digital gesture recognition [5]. Borghetti et al. studied and implemented a finger position measurement system with the aim of providing feedback to the rehabilitation system [6]. They measured the bending angle of finger joints by placing 10 bending resistors on the back of five fingers. To improve the measurement accuracy, they used a piecewise second-order polynomial fitting method to correct the data. Saggio developed a glove embedded with a carbon ink-based array of flex resistor sensors [7].

The IMU is a device that measures the attitude angle (or angular velocity) and acceleration of an object on three axes. There are 3-axis, 6-axis and 9-axis IMUs. Connolly et al. developed and tested a wireless glove embedded with 16 9-axis IMUs to facilitate accurate measurement of finger motions [8]. They experimentally compared the performance of their glove with that of the 5 DT data glove [3]. The results showed that their glove achieved higher accuracy and less overall errors than the 5 DT data glove. In addition, clinical trial results indicated that the glove could provide a reliability within 5°. To simultaneously measure hand kinematics and fingertip force, a sensorized glove combining 18 9-axis IMUs and four force sensors was developed in a study by Lin [9]. Their results showed that the mean absolute errors of the finger joint angles were all less than five degrees. Zou et al. investigated a glove embedded with an IMU to provide real-time monitoring and coaching services for training participants [10]. To evaluate the hand functions of patients suffering strokes, Lin et al. proposed a data glove embedded with 14 6-axis IMUs [11]. The glove has the ability to provide the hand’s acceleration, angular velocities and joint angles. Dai et al. developed a glove monitoring system based on an IMU and an FSR to quantify neurological symptoms during deep-brain stimulation surgery [12].

The optical fiber sensor is a type of sensor in which the light travelling through the fiber will change when any change is measured. Several optical fibers can be used to make data gloves. Huang et al. proposed a data glove by sewing the reduced grapheme oxide-coated fibers onto a textile glove. They carried out a static gesture recognition experiment and a dynamic gesture recognition experiment, and the recognition accuracy rates were 98.5% and 98.3%, respectively [13]. Michiko et al. presented a sensing glove with hetero-core fiber-optic nerve sensors [14]. They measured the optical loss in the fiber to reflect the finger bending angle. Chandan Kumar et al. demonstrated a sensing glove based on a fiber Bragg grating (FBG) sensor [15]. Their results showed that the finger joint angle measurement accuracy was 0.67°. Fujiwara et al. reported a multimode fiber bending transducers based glove to monitor the thumb posture [16]. Jiang et al. developed a glove embedded with an FBG sensor to capture finger motion, wrist rotation and contact force [17].

Except bending resistors, IMUs and optical fiber sensors, magnetic coils, capacitive sensors, and other materials/devices have been used to develop data gloves. Fahn et al. developed a data glove by using five magnetic coils placed on the palmar surface to measure 10 degrees of freedom of a hand [18]. Pan et al. presented a glove with 16 capacitive sensors embedded to capture the hand gesture [19]. In 10 American Sign Language gesture recognition experiments, they got a classification accuracy of 99.7% by using machine learning algorithms and directly processing the code-modulated signals. Lee et al. presented a dynamic finger gesture recognition system by using a data glove embedded with 10 soft sensors [20]. The soft sensors employed in their research were made via direct writing of eutectic Gallium Indium. Kanokoda et al. fabricated a pyrolytic graphite sheet strain sensor based glove to capture hand movements for gesture prediction [21]. Nassour et al. prepared sensors based on a silicone tube and a conductive liquid for the measurement of finger joint angles [22]. Sundaram et al. conducted the research of individual objects indentification and their weight estimation by using a scalable tactile glove and deep convolutional neural networks. There is a sensor array (548 sensors) is assembled on a knitted glove [23].

Despite their ability to monitor and track hand motions, data gloves still suffer from some limitations. Most data gloves based on bending resistance can only qualitatively measure finger bending angles, which is unsuitable for occasions requiring high measurement accuracy. IMU-based data gloves have high measurement accuracy. Most low cost IMU sensors, however, have drift problems. Therefore, complex algorithms should be used to process the data and thereby get better measurement results, which increases the calculation burden. In addition, IMU-based data gloves are susceptible to electromagnetic interference in the environment. Data gloves based on optical fiber sensors are immune to electro-magnetic interference, but they are temperature sensitive.

This paper presents a data glove for capturing hand finger joint angles. 14 sensing units based on a flexible grating strip are placed on the back of the glove to get the angles of distal interphalangeal (DIP) joints, proximal interphalangeal (PIP) joints, metacarpophalangeal (MCP) joints of all fingers. In order to reduce the wiring harness in gloves, a distributed signal processing unit based on STM32 and IIC bus is designed. Response tests and calibration experiments are conducted to verify the feasibility and effectiveness of the designed sensing unit. Grasping tests and gesture recognition experiments are conducted to evaluate the performance of the data glove.

The rest of paper is organized as follows. Section 2 illustrates the design and fabrication of the sensing unit and the data glove. The experiments and results are presented in Section 3. Conclusions are given in Section 4.

## 2. Materials and Methods

### 2.1. Design of Sensing Unit

**Figure 1:**
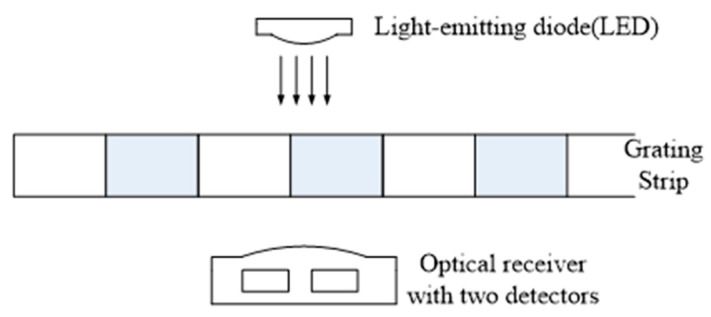
Displacement detection principle based on the grating strip.

**Figure 2:**
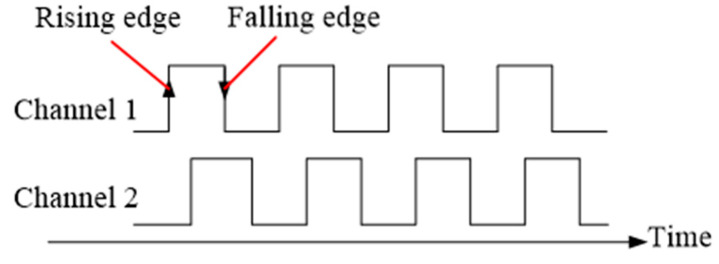
The typical pulse signals output by the optical receiver.

There are several existed solutions for finger joint angle measurement. However, the low cost IMU sensor has a gyro-drift problem, the ordinary optical fiber is sensitive to environmental temperature, and FBG needs a special signal demodulator and is not portable. In this paper, a flexible grating strip is employed as the sensor unit to measure the finger joint angle. There are equidistant parallel opaque stripes in the grating strip. As shown in Figure 1, with the help of a light-emitting diode (LED) and an optical receiver, we can detect the movement of the grating. When light strikes on the opaque strip, the detector outputs a low level since it cannot receive an optical signal. On the contrary, the detector outputs a high level when light strikes on the transparent area. When the detector position is fixed and the grating moves relative to the detector, the detector will output a series of pulse signals. The number of pulses is proportional to the movement of the grating. That is to say, the amount of grating movement can be calculated by counting the output pulse of the detector. Since the two detectors in the optical receivers are placed side by side along the direction of the grating’s movement, one detector will always receive the light signal before the other detector when the movement of the grating is identified. Figure 2 shows a typical pulse signal output by the optical receiver. Based on the description above and the typical pulse signals shown in Figure 2, the moving direction of the grating strip can be determined by detecting the sequence of rising edges of two signals. Based on the identified movement direction, the displacement of the grating strip can be obtained by counting the number of edges in the signal.

In this paper, a flexible grating strip (Seiko Epson Corporation, Japan) with 7 stripes per millimeter and the optical detector module which integrates one LED and two detectors are employed. In the optical detector module, there is a gap between the LED and the optical receiver. When a grating strip moves in the gap, pulses related to displacement information will be output by the optical detector. Since there are 7 strips per millimeter in the grating strip and there are two detectors in the optical detector module, the optical detector module will output two channels of signals which each channel contains 7 pulses when the grating strip moves 1 mm. For each pulse, there are two edges, one of which is rising edge and the other is falling edge. If the rising and falling edges of the pulses are counted for representing the displacement of the grating strip, we can obtain 28 (2 channels × 7 pulses × 2 edges) edges in total when the grating strip moves 1 mm. So, the resolution of displacement detection is 0.036 mm (1 mm/ 28 ≈ 0.036 mm).

**Figure 3:**
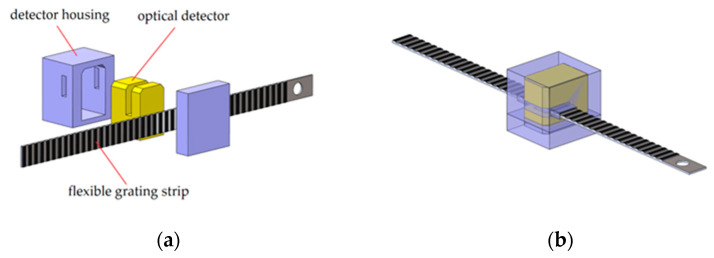
Prototype of the sensing unit. (**a**) Components of the sensing unit; (**b**) Assembly of the sensing unit.

**Figure 4:**
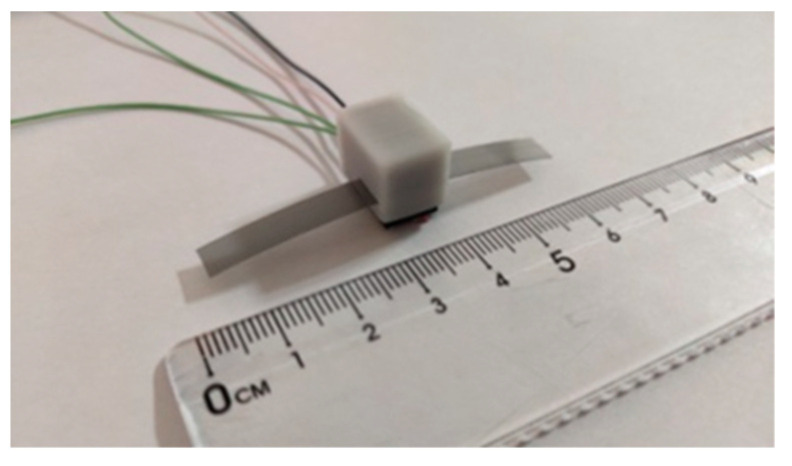
The picture of the designed sensing unit.

Figure 3 shows the prototype of the designed sensing unit. It contains three units: a flexible grating strip, an optical detector, and a detector housing. The optical detector is fixed in the detector housing. A gap on each side of the shell is set to make the grating strip move smoothly. Figure 4 is the picture of the designed sensing unit.

**Figure 5:**
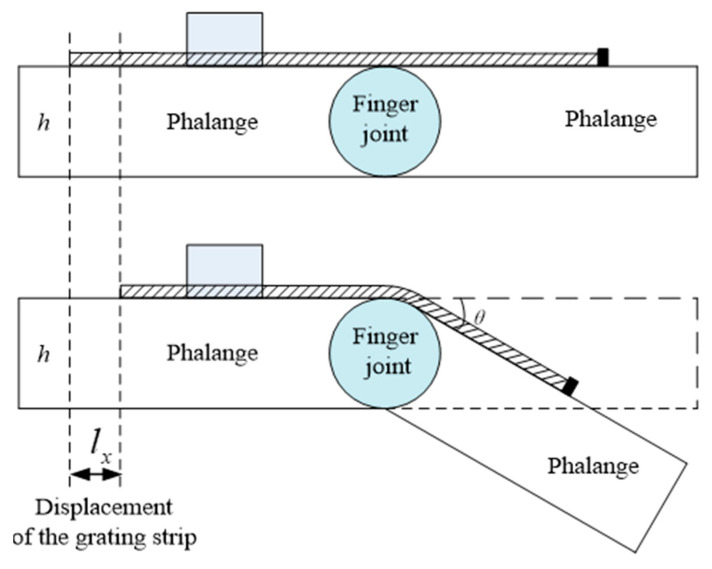
The diagram of conversion from displacement to angle.

Figure 5 shows a schematic diagram of using the sensing unit for finger joint angle measurement. The sensing unit is fixed on the back of the finger. One side of the grating strip is fixed on the back of the finger; the other side is unconstrained and can be moved freely. When the finger bends, the free end of the grating strip will move along the gap in the detector housing. Since the joint angle is considered as 0° under the condition that the fingers are stretched out, the flexion angle, *θ* (in degrees), can be expressed as follows:

(1) $$\theta =\frac{360}{\pi }\times \frac{l_{x}}{h}$$

where *l_x_* is the displacement at the free end of the grating strip, and *h* is the finger’s thickness.

Further, we can take the derivative to get the angular velocity and the acceleration of the finger joint.

### 2.2. Design and Fabrication of a Glove Embedded with Sensing Units

**Figure 6:**
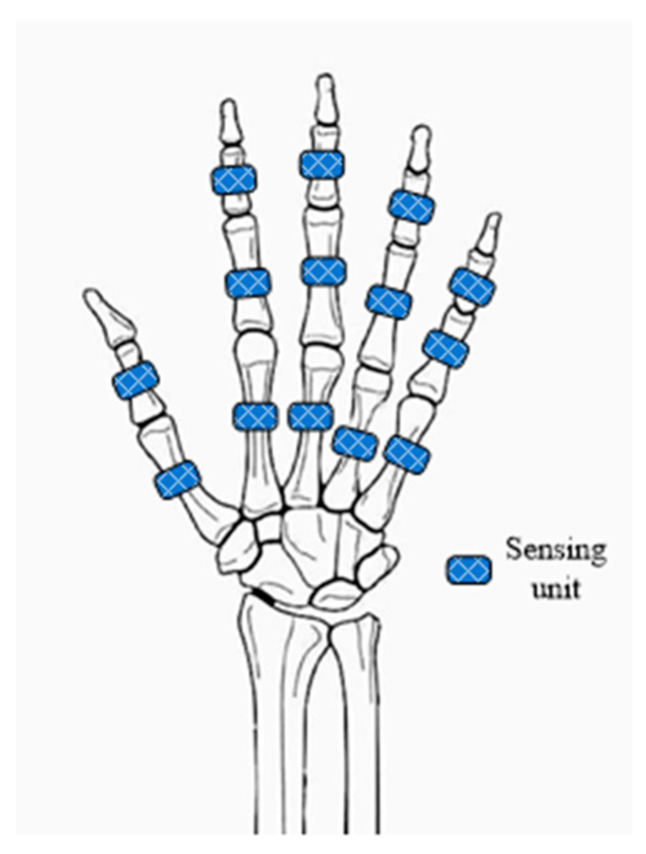
Distribution of sensing units on the back of a hand.

To obtain the information about the bending of each joint of the finger, the configuration of the glove is designed with sensing units, as shown in Figure 6. Fourteen sensing units are arranged on the back of the glove. There is a sensing unit on the back of the middle phalange, proximal phalange and metacarpal of each finger, except for the thumb. For the thumb, two sensing units are distributed on the back of the proximal phalange and metacarpal, respectively. For the grating strip in each sensing unit, one side of the strip is fixed on the back of the glove; the other side is unconstrained and can be moved freely. As a result, the DIP, PIP and MCP joints of index, middle, ring and little fingers and the DIP and MCP joints of the thumb can be captured.

According to the analysis in Figure 5, when the joint is bent by 110°, the displacement of the grating strip is approximately equal to the joint diameter (lx = (110/360)πd ≈ d). Therefore, the collision between the grating and the electronic box can be avoided only if the distance between the two electronic fingers is greater than the joint diameter when preparing gloves. In fact, the length of the phalange is much larger than the diameter of the finger joint, so that the grating in the sensing unit will not collide with the electronic box of the other sensing unit when preparing the glove by simply fixing the electronic box in the middle of the phalange.

**Figure 7:**
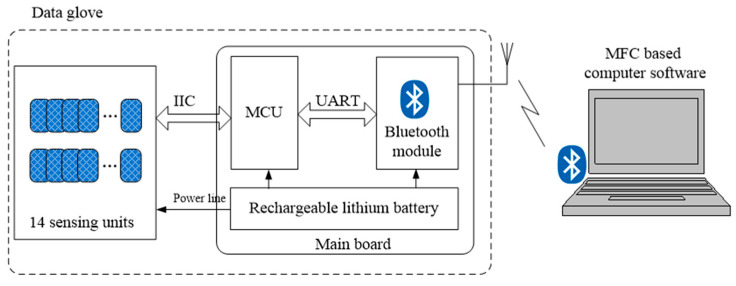
Architecture of the wireless data glove.

Because two channels of signals are related to the joint angle for each sensing unit, and 14 sensing units are distributed on the back of the glove, a large number of wires are needed to connect signals to the glove’s microprocessor unit (MCU). As shown in Figure 7, in order to reduce the number of wires, a distributed signals processing and connection architecture is designed in this paper.

STM32L011D4P6, a low-power, small-sized single-chip microcomputer with the ability to process encoded signals and IIC communication, is employed as the distributed processor. Signals output from each sensing unit are processed first by STM32L011D4P6 and then transmitted to the main board via the IIC bus. Each STM32L011D4P6 on the sensing unit has a specific address. The MCU on the main board collects the angle data of each finger joint according to the address of the slave via the IIC bus. In designing the glove, we measured the speed of the fingers on the keyboard, which are normally four to seven strokes per second for most people. Therefore, the sampling rate of our gloves is set to 15Hz for each sensing unit to ensure that normal finger movements can be captured. The data collected by the main board is sent to the computer via the Bluetooth module. On the computer, a Microsoft foundation class based software is developed to display and record the joint angle data. In practice, there are different requirements for the sampling frequency of data gloves. In applications with sampling frequency requirements within 80 Hz, improving sampling frequency can be achieved by increasing the baud rate of IIC. Limited by IIC communication speed, the current system architecture cannot meet the requirements of relatively very high sampling frequency applications such as haptics (1 kHz). For these applications, using FPGA to process the output signals of each sensing unit is a feasible solution. FPGA has a strong parallel processing ability, which can quickly process the signals of 14 sensing units and significantly reduce the time of data transmission. This architecture can achieve ultra-high sampling frequency. 

**Figure 8:**
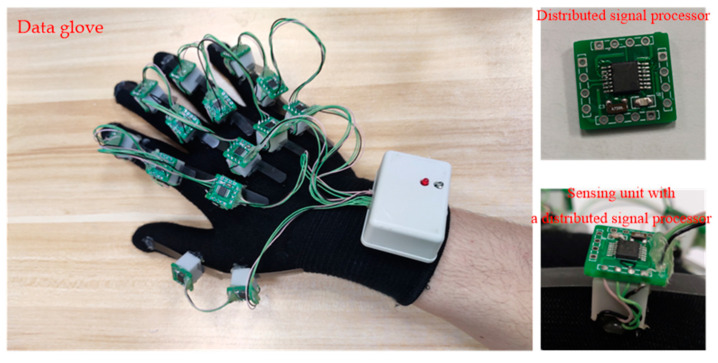
Photo of the designed wireless data glove.

Figure 8 shows the prototype of the designed data glove. It comprises three parts: a fabric glove, 14 sensing units and a main board.

## 3. Experiments and Results

In this section, durability test of grating strip, sensing unit response tests, calibration experiments and glove performance tests are conducted.

### 3.1. Durability Test of Grating Strip

**Figure 9:**
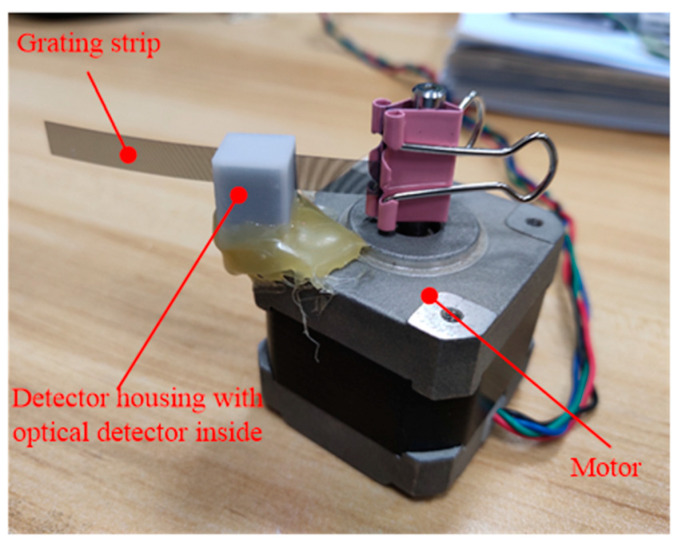
Durability test of grating strip.

Since we do not know the material properties of the grating strips, the experimental was conducted to verify the durability of the grating strip used in the design. As shown in Figure 9, the grating strip and the sensing unit are fixed on the motor output shaft and motor shell, respectively. The motor is controlled to do a reciprocating motion of 120° to simulate the bending of fingers. Two gratings were randomly selected for testing. After the motor reciprocating for 20,000 times, the grating bars did not break. And, there is no obvious wear on the surface of the grating strip, which will not affect its sensing function.

### 3.2. Sensing Unit Response Testing

Two response tests are conducted to evaluate the feasibility of using the designed sensing unit for finger joint measurement.

#### 3.2.1. Response on Wooden Finger Model

**Figure 10:**
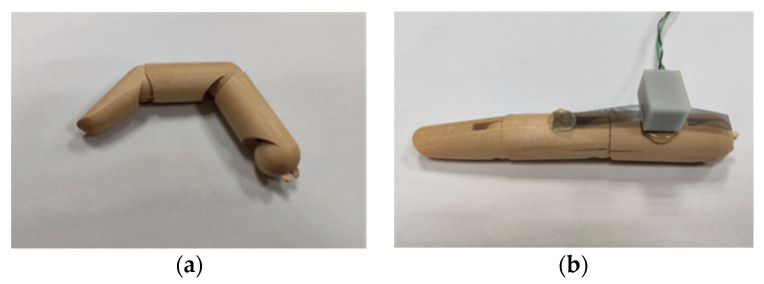
Response testing device. (**a**) Wooden finger; (**b**) Wooden finger with a sensing unit.

The designed sensing unit is installed on the finger model, and the response of the sensing unit is observed by bending the joint of the finger model. Figure 10 shows a photo of the wooden finger model and a photo of the wooden finger model with a sensing unit worn on it. The bottom side of the detector housing is attached to the back of the proximal phalange of the finger model by hot-melt adhesive. One end of the grating strip is also connected to the back of the middle phalange of the finger model by hot-melt adhesive.

**Figure 11:**
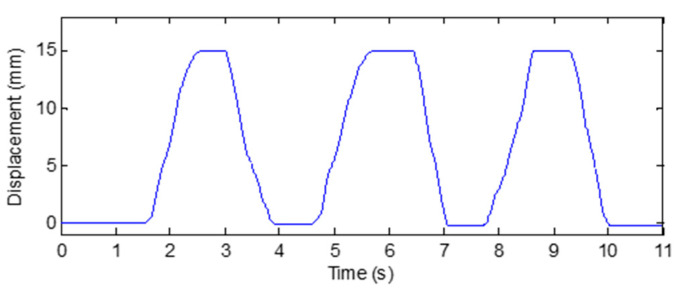
Response of the sensing unit on the wooden finger model.

The displacement of the grating strip is recorded during the reciprocating rotation of the finger’s PIP joint, and the corresponding curve as shown in Figure 11 is obtained. It is found that the displacement of the grating strip always changes between 0 and 16 mm, demonstrating the stable and repeatable response of the grating strip when the finger joint is bent.

#### 3.2.2. Response on Human Finger

**Figure 12:**
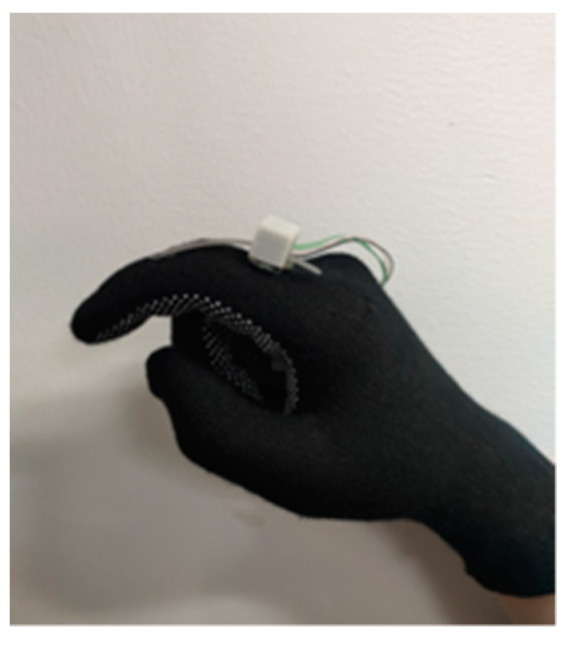
The designed glove with a sensing unit on it.

As shown in Figure 12, the designed sensing unit is installed on the back of the glove. In this test, subjects put on the glove and bend their index fingers. During this process, the displacement of the grating strip is recorded, and the response of the sensing unit on the human finger is obtained. To be specific, this test includes the following four test items. A right hand-dominant healthy subject participates in Test A and Test B. Ten right hand-dominant healthy subjects (four males and six females, aged from 20 to 33) participate in Test C and Test D.

**Test A:** The subject has his index finger flexed and extended several times after wearing the glove with the sensing unit.

**Test B:** The subject orderly bends his index finger at four different angles for a few seconds in each cycle.

**Test C:** Subjects put on the glove with the sensing unit and then bend the PIP joint of their index finger completely 10 times. The displacement of the grating strip when the finger is bent completely is recorded.

**Test D:** This test is similar to Test C except that the glove with the sensing unit is taken off and put on between blocks.

**Figure 13:**
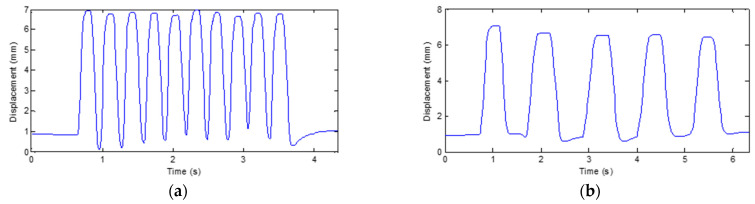
Results of Test A in response testing on human finger. (**a**) Fast flexion and extension; (**b**) Slow flexion and extension.

**Figure 14:**
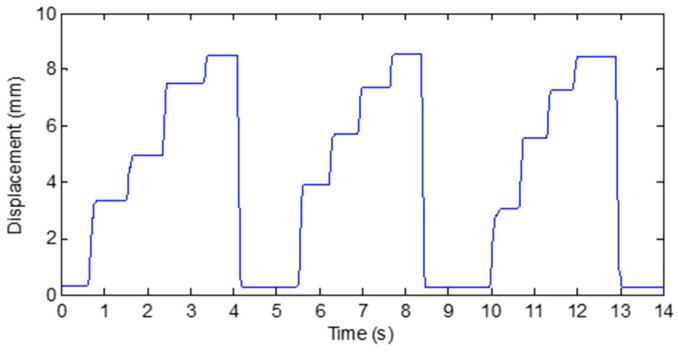
Results of Test B in response testing on a human finger.

Figure 13 and Figure 14 show the results of Test A and Test B, respectively. It can be seen that the sensing unit has the ability to respond quickly and stably to the bending angle of human finger joint.

Table 1 shows the results of Test C and Test D. For each subject, the average value (AV), the root mean square (RMS) and the normalized root mean square (NRMS) of the displacement of the grating strip are calculated when the subject’s index finger changes from the straight state to the completely bent state. The expressions of AV, RMS and NRMS are shown in Equations (2)–(4) respectively.

The results are different due to the distinction of finger size. For all subjects, Test D delivers greater *RMS* and *NRMS* values than Test C. This indicates that, when the glove with a sensing unit is used to measure the finger joint angle, wearing the glove repeatedly will lead to difference between different measuring trails.

(2) $$AV=\frac{1}{N}\sum_{i=1}^{N}P_{i}$$

(3) $$RMS=\sqrt{\frac{\sum_{i=1}^{N}{\left(AV-P_{i}\right)}^{2}}{N-1}}$$

(4) $$NRMS=\frac{RMS}{AV}$$

where, *P_i_* is the displacement of *i*th trail, and *N* is the number of trails.

### 3.3. Calibration Experiments

#### 3.3.1. Calibration of Sensing Unit on Wooden Finger Model

Since the finger joint does not come with an ideal structure as shown in Figure 5, it will lead to an error between the angle calculated by using Equation (1) and the real angle. To obtain the bending angle of the finger joint accurately, it is necessary to calibrate the designed sensing units. In this paper, an MPU6050-based gyroscope module, JY61P, is employed as a reference sensor to carry out calibration experiments. An attitude solver and a dynamic Kalman filtering algorithm are integrated inside the module, which can output the Euler attitude angle in the Cartesian coordinate system under a dynamic environment with an accuracy of 0.05° and a stability of 0.01°.

**Figure 15:**
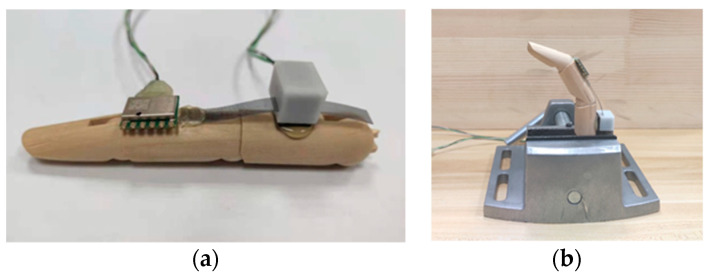
Calibration experiment platform. (**a**) Wooden finger model with a sensing unit and a gyroscope; (**b**) Wooden finger on the flat tongs.

**Figure 16:**
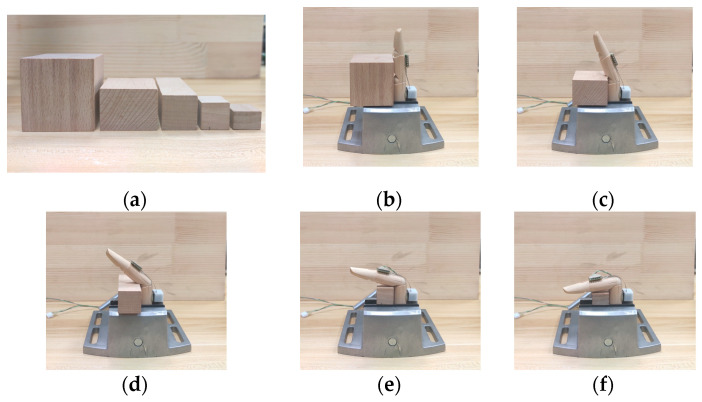
Five calibration points set by five wooden blocks with different heights. (**a**) Wooden blocks used to set bending angles; (**b**) Bending angle is 0°; (**c**) Bending angle is 21.53°; (**d**) Bending angle is 48.07°; (**e**) Bending angle is 72.25°; (**f**) Bending angle is 96.18°.

Take the calibration of the PIP of the wooden finger model as an example. As shown in Figure 15, the wooden finger model on which the designed sensing unit and gyroscopes are placed is held by flat tongs. During calibration, several fixed bending angles should be selected as the calibration points. In this paper, five wooden blocks with different heights shown in Figure 16a are employed to set the bending angle of the finger joint. As shown in Figure 16b–f, with the wooden block pressed against the proximal phalanx, the PIP joint is rotated until the middle phalange is in contact with the block, so that a specified bending angle can be determined. The measurement results of gyroscope show that, with these five blocks, the bending angle of the finger can be set to 0°, 21.53°, 48.07°, 72.25°, and 96.18°, respectively. In the experiment, the bending angle of the finger is set from minimum to maximum using these wooden blocks, and the displacement of the grating is recorded. Then the angle of the finger is set in reverse from low to high. This process is repeated 6 times, and the recorded data is shown in Table 2 and Table 3.

- Sensitivity

Sensitivity describes the ability of a sensor to respond to changes in finger bending angle. The greater the sensitivity of the sensor, the greater the variation of the output. The average value of the six forward strokes and reverse strokes are calculated as the displacement of the grating strip at each calibration point. And, the linear relationship between finger joint angle and the displacement of the grating strip can be obtained by the least square method as follows:

(5) y=0.15x+0.13

where *y* is the displacement of the grating strip and *x* is the finger joint angle. This equation indicates that the sensitivity of the designed finger joint angle sensor is 0.15 mm/°.

- Nonlinearity

Nonlinearity is used to describe the maximum deviation between the actual transfer function of the sensor and the approximate line. It can be expressed as follows:

(6) $$\xi_{L}=\frac{\left|\Delta Y_{L,\max}\right|}{Y_{F.S}}\times 100\%$$

where *Y_F.S_* is the full scale output of the sensor calculated according to the fitting equation, and △Y*_L,max_* is the maximum difference between the actual average output at each calibration point and the theoretical output of the fitting equation. 

In this paper, the nonlinearity of the designed joint angle sensor is 1.31%.

- Hysteresis error

Hysteresis error is used to describe the degree to which input-output (finger bending angle-displacement of the grating strip) curves do not coincide of the sensor during the forward and reverse travel. It can be expressed as follows:

(7) $$\xi_{H}=\frac{\left|\Delta Y_{H,\max}\right|}{Y_{F.S}}\times 100\%$$

where △*Y_H_*_,max_ is the maximum difference between the average calibration characteristics of the forward stroke and the reverse stroke.

In this paper, the hysteresis error of the designed joint angle sensor is 0.86%.

- Repeatability error

Repeatability error is used to describe the random error. It can be expressed as follows:

(8) $$\xi_{R}=\frac{\lambda \cdot \sqrt{\frac{1}{2}(\frac{1}{m}\sum_{i=0}^{m}S_{I,i}^{2}+\frac{1}{m}\sum_{i=0}^{m}S_{D,i}^{2})}}{Y_{F.S}}\times 100\%$$

where *λ* is the coefficient of fiducial probability, *m* is the number of calibration points, and *S_I,i_* and *S_D,i_* are the standard deviations of positive and negative stroke calibration data at the *i*th calibration point, respectively.

In this paper, the repeatability error of the designed joint angle sensor is 0.57% when the fiducial probability is about 97.5% (*λ* equals 3).

- Comprehensive precision

Comprehensive precision is an estimation of actual measurement error by using nonlinearity, hysteresis error and repeatability error. It can be calculated in several ways. In this paper, the comprehensive precision is calculated to be 1.67% using the following expression:

(9) $$A=\sqrt{\xi_{L}^{2}+\xi_{H}^{2}+\xi_{R}^{2}}$$

#### 3.3.2. Fast Calibration of Sensing Unit on Human Finger

Calibration is a conventional method to improve the accuracy of finger joint angle measurement. Since the fingers of different users are significantly different, and the results of experiment Section 3.2.2 show that wearing the glove repeatedly will lead to measurement difference, it is necessary to conduct separate calibrations for different users to maximize the accuracy of finger joint angle measurement. However, this dramatically reduces the ease of using data gloves, as calibration is a complex professional operation. This is also bound to increase the time consumption in using a data glove.

To reduce the difference of different individuals on the measurement accuracy and avoid complex calibration procedures, a convenient data processing method is proposed as follows:

(10) $$\theta =\frac{l_{x}}{l_{\max}-l_{\min}}\times \theta_{range}$$

where *θ_range_* is the preset maximum finger joint angle, *l_x_* is the displacement at the free end of the grating strip, and *l*_min_ and *l*_max_ are the displacements at the free end of the grating strip when the finger is at full extension and flexion, respectively. 

Since *l*_min_ and *l*_max_ are different for both different individuals and different finger joints, the following steps are performed to get these parameters.

Step 1: Calculating the average displacement of a grating strip in a time window.

(11) $$Ave(t)=\frac{1}{N}\sum_{n=0}^{N-1}l(t-n)$$

where *l*(*t*) is the displacement of the grating strip at the current sampling moment, *l*(*t* − *n*) is the displacement at the sampling moment *n* before the current one, and *N* is the number of sampling points.

Step 2: Updating data.

(12) $$l_{\min}=\{\begin{array}{c} l_{\min}\begin{array}{cc} & ,\begin{array}{cc} & l_{\min}\leq Ave(t) \end{array} \end{array}\\ Ave(t),\begin{array}{cc} & l_{\min}>Ave(t) \end{array} \end{array}$$

(13) $$l_{\max}=\{\begin{array}{c} l_{\max}\begin{array}{cc} & ,\begin{array}{cc} & l_{\max}\geq Ave(t) \end{array} \end{array}\\ Ave(t),\begin{array}{cc} & l_{\max}<Ave(t) \end{array} \end{array}$$

According to the parameter updating process mentioned above, when the user puts on the gloves and turns on the power switch, only one full extension and one full flexion can complete updating the parameters, and then the finger joint angles can be measured continuously.

If a restriction applies to using the glove, that is, the fingers of the gloved hand are required to be in a straight state before the glove signal collector is powered on, the signal processing process can be simplified into Equation (14). Because the detector detects a displacement of 0 the moment the power is turned on, the finger joint angle when the finger is straight is 0°. In practice, this restriction can be achieved easily by opening the hand and placing it against a flat surface.

(14) $$\theta =\frac{l_{x}}{l_{\max}}\times \theta_{range}$$

In this paper, the *θ_range_* values of the MCP and PIP of the thumb are preset to 80° and 90°, respectively. For the other fingers, the *θ_range_* values of the MCP, PIP and DIP are preset to 90°, 110°, and 30°, respectively. 

As described above, we first record the displacement of the grating strip when the subjects’ fingers are completely bent during the experiment, and then repeat Test C as mentioned in Section 3.2.2. The results obtained when subjects bend their index PIP joint 10 times are shown in Table 4. We can see that for all the ten subjects, the RMS of PIP angle measurement is better than 3.29° after the results are processed with fast calibration.

To validate the finger joint angle obtained by the designed sensing unit and fast calibration method, a comparative experiment is carried out. During the experiment, subject orderly bend their index fingers at four different angles. And a camera records the photos of the fingers in different positions. Then, Image J is introduced to measure the finger joint angle. Table 5 shows the comparison of measurement results of a subject. Compared with Image J, the maximum angle deviation of the designed sensing unit is 4.14° (3.76% FS). It indicates that an acceptable finger joint angle measurement error can be obtained by using fast calibration method.

As listed in Table 6, four different solutions are compared with our proposed grating strip based solution. It can be found that all the solutions, especially IMU and FBG based solutions, listed in the table can provide a good performance while measuring the joint angle. However, FBG preparation, signal demodulator greatly increase the cost. In addition, the volume and weight of FBG signal demodulator with multiple measurement points is large and heavy, so it is difficult to realize the portable preparation of a data glove. For low cost IMU based solutions, drift and susceptible to electromagnetic interference also limits their applications. For the solution proposed in this paper, the measurement precision and cost are taken into account. It is not sensitive to electromagnetic interference and does not require complex algorithms to overcome the drift problem in IMU.

### 3.4. Glove Performance Tests

Two tests are conducted to evaluate the performance of the designed glove.

#### 3.4.1. Grasping Tests

**Figure 17:**
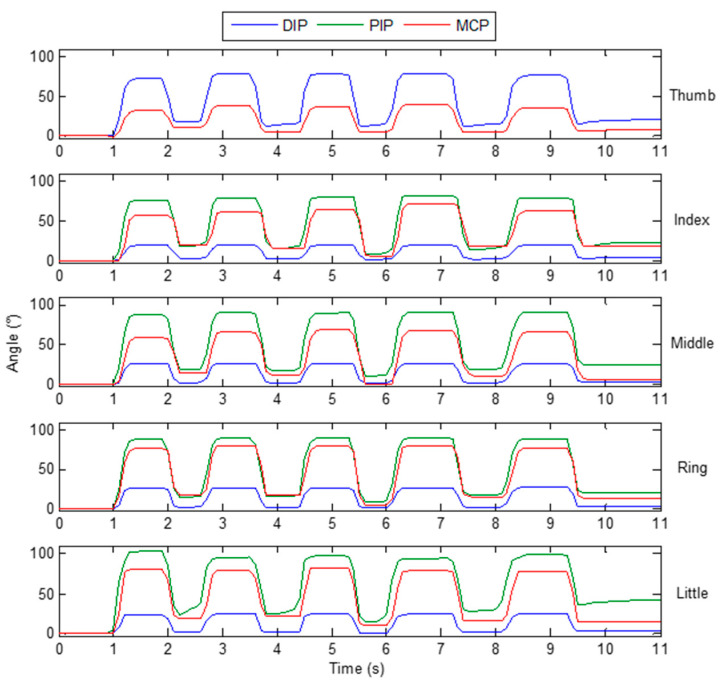
Finger joint angles when a subject makes a continuous grasping motion.

A subject is asked to make a continuous grasping motion with the designed glove on his hand. Figure 17 shows the bending angle changes of each finger joint during this process. The results show that the 14 finger joint angles can be captured by the designed data glove. And, the captured results of continuous grasping motion show a good consistency. 

#### 3.4.2. Abduction and Adduction Test

**Figure 18:**
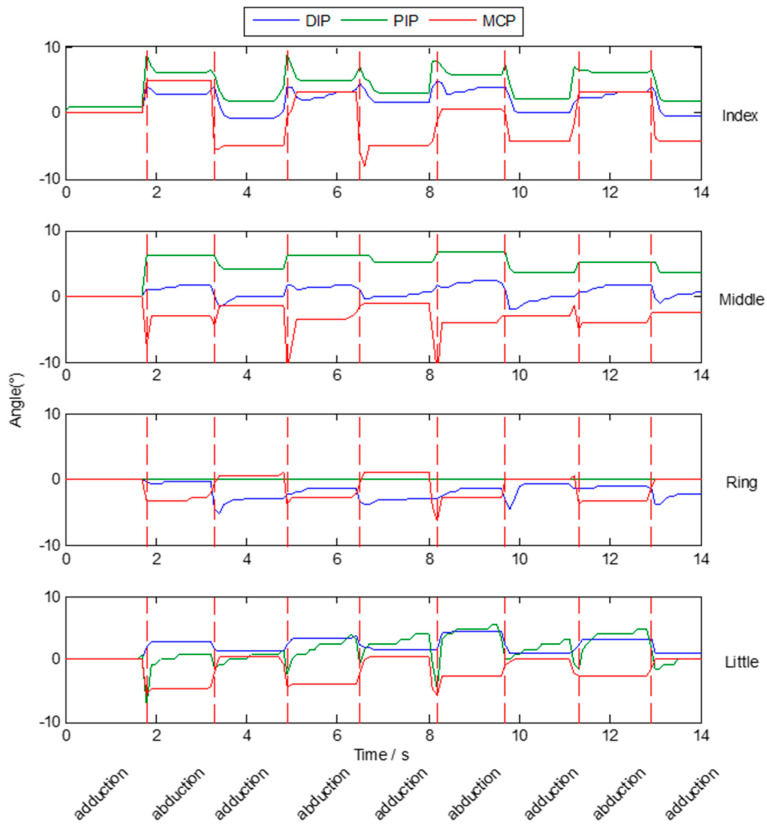
Finger joint angles when a subject makes a continuous abduction and adduction motion.

Besides flexion and extension, abduction and adduction are also important for a data glove. The prototype designed in this paper only pays attention to the detection of the flexion and extension of the finger joint. Since there are no sensor units in the glove to detect abduction/adduction finger movement, the actual abduction/adduction movement could not be measured currently. In this section, the purpose of abduction and adduction test is to illustrate the influence of abduction/adduction finger movement on finger bending angle measurement. The abduction/adduction movement of the thumb is often accompanied by flexion/extension of the joint. Here we only tested the movement of the other four fingers. Since abduction /adduction movement will cause an extra angle change with bended finger, the influence of abduction /adduction movements are tested without any flexion/extension motions. During the experiment, a subject is asked to place his palm flat on a table. Figure 18 shows the change of finger joint angles when a subject makes a continuous abduction and adduction motion. The results indicate that the deviations of each finger joint angles when the fingers fully abduction compared with when the fingers fully adduction are less than 10°.

#### 3.4.3. Gesture Recognition based on the Designed Data Glove and Neural Network

**Figure 19:**
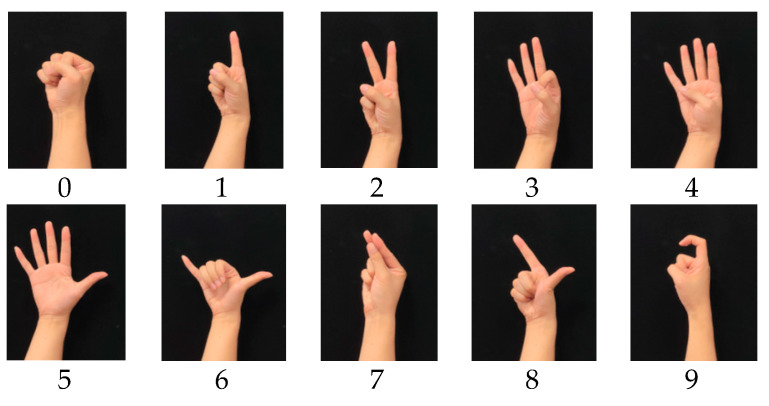
Digital gestures for 0 to 9.

**Figure 20:**
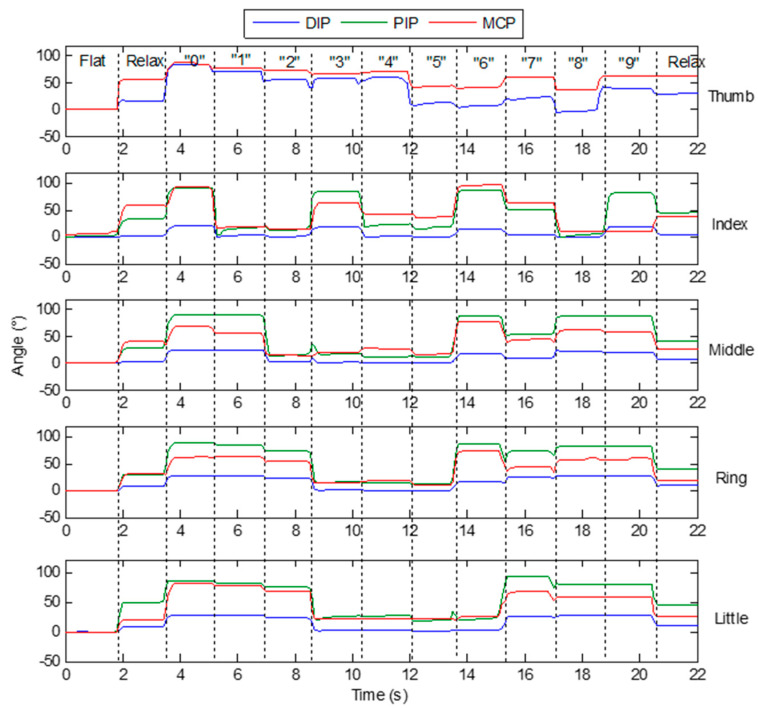
Finger joint angles when a subject continuously makes different digital gestures.

Gestures are a common way for people to communicate and convey information in daily life. A lot of research has been done on gesture recognition based on finger bending information [5,19,20,21,22]. In this paper, static gesture recognition based on the designed data glove and neural network is conducted to verify the effectiveness of the designed glove in daily application. As shown in Figure 19, ten static gestures that represent numbers 0 to 9 are selected as the recognition targets. Figure 20 shows the finger joint angles when a subject continuously makes different digital gestures.

**Figure 21:**
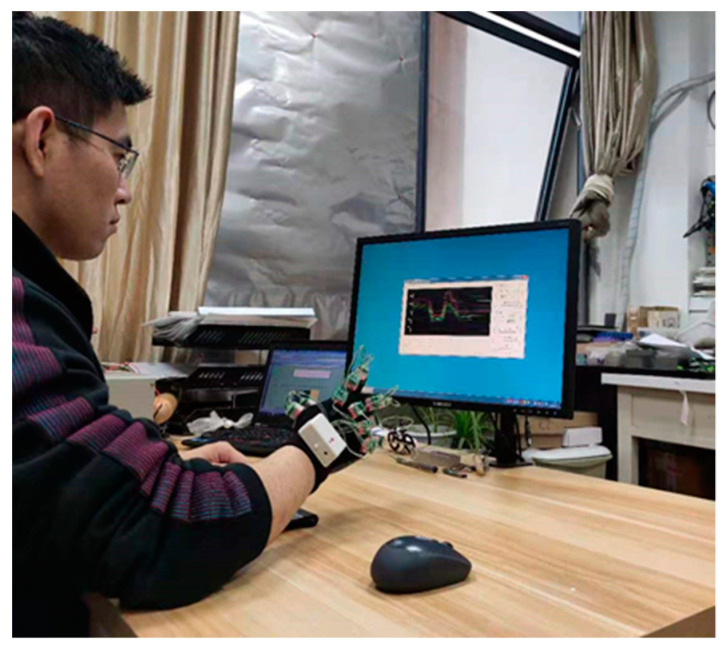
Experimental scene of digital gesture recognition.

The ten subjects in Experiment Section 3.2.2 participate in the digital gesture recognition experiment. Figure 21 shows the experimental scene. Subjects sit in front of a computer with their right forearms on the table and data gloves on their right hands. Subjects are allowed five minutes to familiarize themselves with the experiments beforehand. In the experiments, they make gestures 100 times as instructed in the computer software. The instruction for each gesture appears 10 times at random. During this process, the finger bending data collected by the data glove and the gesture are recorded synchronously by the computer software.

After data collection, GRNN is employed as the recognition model to identify the digital gestures. In this paper, two different tests are conducted as follows.

**TestA:** All the subjects’ data is pooled together for model training and testing. 50% of the data is randomly selected for network training and the remaining 50% for testing. The experiments are repeated five times, and the average result of the five experiments is used as the final gesture recognition result.

**Test B:** All data of a subject is used for network training, and all data of the remaining subjects are used for network testing.

**Figure 22:**
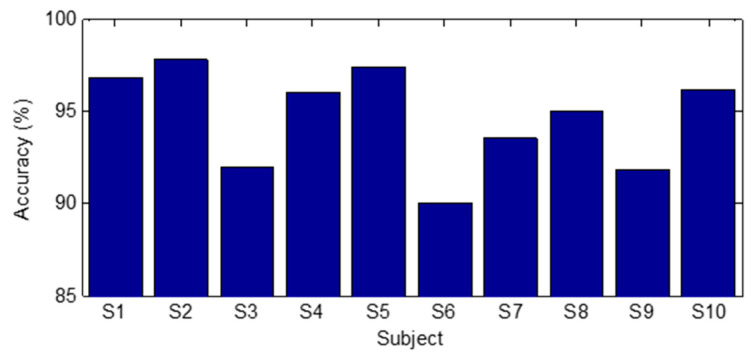
Results of Test B in digital gesture recognition experiment.

Table 7 shows the confusion matrix of the gesture recognition experiment for Test A. The overall recognition accuracy is 98.8%. Figure 22 shows the results for Test B. When the network trained by the data of one subject is used to recognize the gestures of other subjects, the recognition accuracy is between 90% and 97.8%. It indicates that a network trained with data from a single subject can be used to recognize the gestures of other subjects.

## 4. Conclusions

In this paper, a novel wireless data glove based on a flexible grating strip is proposed for capturing finger joint angles. Fourteen sensing units based on the flexible grating strip are designed and distributed on the back of a fabric glove so that the DIP, PIP, and MCP joint angles of all fingers can be measured. Based on STM32 and IIC bus, a signal processing and transmission system is constructed for the data glove. At the same time, the wireless portability of data glove is realized by Bluetooth communication. Sensing unit response, calibration, grasping and static digital gesture recognition experiments are carried out to evaluate the performance of the designed sensing unit and data glove. The experimental results show that the sensing unit can effectively reflect the bending angle of the finger joint; when measuring the joint angle of the wooden finger model, the designed sensing unit can deliver a sensitivity of 0.15 mm/°, a nonlinearity of 1.31%, a hysteresis error of 0.86%, a repeatability error of 0.57%, and a comprehensive precision of 1.67%. In the gesture recognition experiment, the overall recognition accuracy is 98.8% and the individual cross recognition accuracy is more than 90%, indicating that the designed glove can perform digital gesture recognition tasks efficiently.

For the next step, we will use the designed glove for dynamic gesture recognition research and further explore other applications of the designed data glove in daily life.

## Figures and Tables

**Table 1:** Results of Test C and Test D.

Subject	Test C			Test D		
	AV (mm)	RMS (mm)	NRMS (%)	AV (mm)	RMS (mm)	NRMS (%)
S1	12.44	0.09	0.71	12.02	0.18	1.46
S2	12.83	0.19	1.48	11.79	0.50	4.26
S3	10.59	0.17	1.61	10.75	0.44	4.12
S4	12.71	0.06	0.46	13.14	0.66	4.99
S5	11.18	0.15	1.38	12.26	0.54	4.39
S6	8.84	0.26	2.98	8.66	0.51	5.90
S7	9.82	0.29	2.95	10.23	0.56	5.47
S8	9.51	0.11	1.16	9.33	0.32	3.43
S9	10.2	0.24	2.35	12.02	0.49	4.08
S10	11.35	0.19	1.67	11.78	0.61	5.18

**Table 2:** Calibration experimental data.

Bending Angle (°)	Displacement of the Grating Strip (mm)					
	First Cycle		Second Cycle		Third Cycle	
	Positive	Negative	Positive	Negative	Positive	Negative
0	0	0.036	0.036	0.036	0.036	0.036
21.53	3.500	3.571	3.500	3.607	3.536	3.607
48.07	7.107	7.143	7.107	7.214	7.143	7.25
72.25	10.857	10.857	10.893	10.857	10.821	10.821
96.18	14.464	14.464	14.500	14.500	14.500	14.500

**Table 3:** Calibration experimental data (continued).

Bending Angle (°)	Displacement of the Grating Strip (mm)					
	Fourth Cycle		Fifth Cycle		Sixth Cycle	
	Positive	Negative	Positive	Negative	Positive	Negative
0.00	0.036	0.036	0.036	0.036	0.036	0.036
21.53	3.500	3.643	3.429	3.643	3.464	3.607
48.07	7.143	7.214	7.143	7.214	7.107	7.214
72.25	10.857	10.857	10.821	10.714	10.893	10.857
96.18	14.464	14.464	14.464	14.464	14.464	14.464

**Table 4:** Results of fast calibration.

Subject	*l*_max_ (mm)	RMS (mm)	RMS (°)	Subject	*l*_max_ (mm)	RMS (mm)	RMS (°)
S1	12.50	0.09	0.78	S6	8.57	0.25	3.15
S2	13.43	0.20	1.64	S7	9.04	0.27	3.29
S3	11.21	0.18	1.76	S8	9.96	0.10	1.1
S4	13.00	0.06	0.51	S9	9.79	0.22	2.42
S5	11.79	0.16	1.53	S10	11.75	0.19	1.82

**Table 5:** Measurement results of Image J and the designed sensing unit.

Angle from Image J (°)	Angle from the Designed Sensing Unit (°)	Deviation (°)	Deviation (% FS)
35.65	39.79	−4.14	3.76
55.15	55.05	0.10	0.09
68.56	67.02	1.54	1.40
90.19	87.21	2.98	2.71

**Table 6:** Comparison of proposed finger joint angle measurement solution and others solutions.

Publications	Type of Sensor	Deviation of Finger Joint Angle (°)
Chan et al. [24]	Flexible piezoelectric sensor	5
Gentner R et al. [25]	Bending resistor	4.96
Da Silva et al. [26]	Fiber Bragg grating sensor	2
Kortier al. [27]	IMU	1.1
Proposed in this paper	Grating strip	4.14

**Table 7:** Confusion matrix of Test A in gesture recognition experiment.

Accuracy (%)		Recognized Gestures									
		1	2	3	4	5	6	7	8	9	0
Actual Gestures	1	100.00	0.00	0.00	0.00	0.00	0.00	0.00	0.00	0.00	0.00
	2	0.00	96.79	0.43	0.92	0.00	0.00	0.00	0.00	1.41	0.44
	3	0.00	0.48	97.69	0.00	0.00	0.00	0.00	0.00	0.89	0.94
	4	0.00	0.50	0.00	99.50	0.00	0.00	0.00	0.00	0.00	0.00
	5	0.00	0.00	0.00	0.00	100.00	0.00	0.00	0.00	0.00	0.00
	6	0.00	0.00	0.00	0.00	0.00	100.00	0.00	0.00	0.00	0.00
	7	0.48	0.00	0.00	0.00	0.00	0.00	98.62	0.00	0.00	0.90
	8	0.00	0.00	0.00	0.00	0.00	0.43	0.00	99.57	0.00	0.00
	9	0.00	0.43	0.00	0.00	0.00	0.00	0.00	0.00	99.10	0.47
	0	0.00	0.00	0.00	0.00	0.00	0.48	1.42	0.00	0.98	97.13

## Data Availability

The data presented in this study are available in article.
